# Management of Chagas Disease in Primary Health Care: Challenges Faced by Physicians in Areas with Higher and Lower Endemicity in Brazil

**DOI:** 10.1007/s10900-026-01595-6

**Published:** 2026-07-23

**Authors:** Andréia Brito de Souza, Denise Alves Guimarães, Renata Fiuza Damasceno, Mayra Domingues Cardoso, Ariela Mota Ferreira, Clareci Silva Cardoso, Ester Cerdeira Sabino, Desirée Sant´Ana Haikal

**Affiliations:** 1 Graduate Program in Health Sciences, State University of Montes Claros, Prof. Braga Avenue, s/n, Vila Mauriceia, Montes Claros, 39401-089 Minas Gerais Brazil; 2 School of Medicine, Federal University of São João del-Rei, Divinópolis, Minas Gerais Brazil; 3 Regional Health Superintendence of Divinópolis, Minas Gerais State Health Department (SES-MG), Divinópolis, Minas Gerais Brazil; 4 State University of Montes Claros, Montes Claros, Minas Gerais Brazil; 5 Institute of Tropical Medicine, University of São Paulo, São Paulo, São Paulo, Brazil

**Keywords:** Chagas Disease, Primary Health Care, Physicians, Continuing Education, Qualitative Research

## Abstract

To analyze Primary Health Care physicians’ perceptions of the challenges involved in managing Chagas disease in areas with higher and lower endemicity. This qualitative study was conducted in three municipalities in the state of Minas Gerais, Brazil. Four focus groups involving 27 physicians were carried out. Discussions were guided by a semi-structured script based on the Clinical Protocol and Therapeutic Guidelines for Chagas Disease, audio-recorded, transcribed verbatim, and analyzed using thematic content analysis according to Bardin, with independent double coding. The findings revealed gaps in professional training, predominance of theoretical teaching, lack of continuing education activities, and limited knowledge of the Clinical Protocol and Therapeutic Guidelines. Low clinical suspicion, uncertainties regarding notification of chronic Chagas disease, and the association of antiparasitic treatment primarily with the acute phase were also identified. In municipalities with higher endemicity, low rates of serological testing requests were observed, whereas in the municipality with lower endemicity, Chagas disease was less frequently considered among diagnostic hypotheses. These findings indicate that educational, clinical, and organizational weaknesses contribute to maintaining the invisibility of Chagas disease within Primary Health Care and that higher local endemicity alone does not ensure its incorporation into routine work processes. The results highlight the importance of continuing education, stronger integration between Primary Health Care and Health Surveillance, and the incorporation of clinical guidelines to enhance the capacity of Primary Health Care services to recognize, diagnose, and provide follow-up care for individuals affected by Chagas disease.

**Supplementary Information:**

The online version contains supplementary material available at https://doi.org/10.1007/s10900-026-01595-6.

## Introduction

Chagas disease (CD) predominantly affects low-income populations with limited access to health services [1]. Minas Gerais (MG), a Brazilian state endemic for CD, accounts for the highest number of disease-related deaths in the country [2, 3]. Approximately 30% of these deaths occur in the Northern Minas Gerais and Jequitinhonha Valley regions, two of the state’s 12 health regions [3]. In addition, these areas have some of the poorest socioeconomic indicators in MG [4]. Northern Minas Gerais is characterized by limited access to health services, which contributes to low clinical suspicion and underdetection of chronic CD cases [5, 6]. A cohort study conducted in this region found that 72% of participants either did not receive medical follow-up or received it irregularly [5].

In this context, Primary Health Care (PHC) plays a strategic role in the diagnosis, treatment, and follow-up care of individuals with CD. In Brazil, the Unified Health System (Sistema Único de Saúde—SUS) provides publicly funded, universal, comprehensive, and free health care [7], with PHC serving as the main entry point to the health system and coordinating patient care [8]. National guidelines recommend that individuals with CD receive longitudinal follow-up at this level of care, including periodic medical consultations and laboratory examinations to monitor disease progression [9]. In addition, antiparasitic treatment with Benznidazole (BZN), recommended as the first-line therapy, can be provided within PHC for eligible individuals [9, 10].

Physicians working in PHC play a key role in the diagnosis, treatment, and follow-up care of individuals with CD [9]. Previous studies have identified several challenges related to the management of CD in PHC, highlighting the need to investigate how these challenges are experienced in clinical practice [11]. Despite the relevance of this topic, studies exploring PHC physicians’ perceptions of the challenges involved in managing CD across epidemiological settings with different levels of endemicity remain scarce. Therefore, this study aimed to analyze the perceptions of physicians working in PHC in the state of MG regarding the challenges involved in the management of CD in areas with higher and lower endemicity.

## Methods

### Ethics Statement

This study was conducted in accordance with Brazilian regulations and the ethical principles established by Resolution No. 466/2012, which governs research involving human participants [12]. The study was approved by the Research Ethics Committee of the State University of Montes Claros (approval No. 5.958.798/2021). Written informed consent was obtained from all participants prior to data collection.

### Study Design

This qualitative study [13] employed focus groups (FG) as the data collection method [14], as this approach was considered particularly appropriate for the study objectives. FG are a form of group interview that uses interaction among participants to generate data and insights that may be difficult to obtain in other contexts. The underlying premise of this method is that collective discussion promotes reflection and clarification of viewpoints that might be less accessible through individual interviews, which tend to provide a more individualized perspective of reality. In addition, the dynamic nature of FG enables the collective construction of meanings through the exchange of experiences among participants [15]. The study followed the Consolidated Criteria for Reporting Qualitative Research (COREQ) guidelines [16].

This study is part of the first phase (situational diagnosis) of a larger implementation science project developed by the SaMi-Trop consortium, the Implementa-Chagas/SaMi-Trop Project [17]. The São Paulo-Minas Gerais Center for Chagas Disease Treatment (SaMi-Trop) is a multicenter initiative involving researchers from four Brazilian public universities.

### Study Setting

The study was conducted in three municipalities in the state of MG, Brazil, including two small municipalities and one medium-sized municipality. Municipality 1 is located in Northern Minas Gerais and has approximately 4,500 inhabitants, an economy based on agriculture and services, a Human Development Index (HDI) of 0.6, and 100% PHC coverage [3, 4]. Municipality 2, also located in Northern Minas Gerais, has approximately 7,600 inhabitants, an economy based on agriculture and services, an HDI of 0.6, and 100% PHC coverage [3, 4].

Municipality 3 is located in the Central-West region of MG and has approximately 73,600 inhabitants, an economy primarily based on the service sector, an HDI above 0.7, and PHC coverage exceeding 73% of the population [3, 4]. Unlike the other municipalities, this municipality has lower endemicity of CD among its native population. It was included in the study because of the high frequency of migration from areas with higher endemicity. Although the entire state of MG is considered endemic for CD, for analytical purposes, Municipalities 1 and 2 were classified as areas with higher endemicity, whereas Municipality 3 was classified as an area with lower endemicity.

### Study Participants

All physicians working in PHC in the selected municipalities were eligible to participate in the study. Exhaustive sampling was employed, consisting of the inclusion of all members of the study population of interest [18]. The study population comprised 4, 2, and 21 physicians in Municipalities 1, 2, and 3, respectively. Invitations to participate were facilitated by the Municipal Health Departments, and no physician declined participation in any of the municipalities.

### Data Collection

The FG were conducted during the second half of 2022. One session was held in each of the two higher-endemicity municipalities (HEM). In the lower-endemicity municipality (LEM), two sessions were conducted because of the larger number of physicians, comprising 11 and 10 participants, respectively. A semi-structured guide was used in all FG. The guide was developed by the research team based on a literature review and on the recommendations of the Clinical Protocol and Therapeutic Guidelines (CPTG) for CD [9]. Each session began with the following guiding question: “What is the reality you face regarding CD in this municipality? Please tell us about your experiences.” Subsequently, specific questions were introduced, and the topics and subtopics addressed are presented in Table 1.

**Table 1:** Topics and subtopics included in the focus group discussion guide

Guiding question: “What is the reality you face regarding CD in this municipality? Please tell us about your experiences.”	
**Topics**	**Subtopics**
*Suspicion and Diagnosis* *Treatment and Follow-up*	• Academic training/professional updating • Municipal epidemiological context • Work processes o serological testing o antiparasitic treatment o referrals • Barriers and facilitators

The FG were moderated by a member of the SaMi-Trop research group with experience in qualitative research, with support from two observers. The moderator had no prior relationship with the participants and did not hold any institutional position that could have influenced their responses. Sessions were conducted in rooms within municipal health facilities that ensured privacy and provided appropriate conditions for group discussions. At the beginning of each session, the objectives of the study were presented. Each focus group continued until all topics included in the guide had been thoroughly discussed and explored by the participants.

All sessions were audio-recorded using two digital recorders and transcribed verbatim by the research team. To ensure participant confidentiality, each participant was assigned an identification code consisting of the letter “P” (participant), followed by a sequential number and the acronyms HEM or LEM, corresponding to higher- and lower-endemicity areas, respectively.

### Data Analysis

Data were analyzed using content analysis as proposed by Bardin [19]. Initially, a pre-analysis phase was conducted, involving repeated readings of all transcribed material to achieve familiarization with the data. Subsequently, the material was explored through the identification, coding, and grouping of meaning units into thematic categories developed inductively from the empirical data. Finally, the results were processed and interpreted in order to identify relevant meanings and patterns.

The categorization process was conducted independently by two researchers. Discrepancies were discussed until consensus was reached, with the participation of two additional researchers in the analytical review process. Representative excerpts from each category were selected to illustrate the findings.

## Results

Four focus group sessions, each lasting approximately 90 min, were conducted, involving a total of 27 participants, including 6 physicians from the HEM and 21 from the LEM. The mean age of participants was 39.17 ± 6.94 years in the HEM and 30.38 ± 4.95 years in the LEM. Regarding professional training, only one physician from the LEM had completed a medical residency program, specifically in internal medicine. All participants were employed under temporary contracts. Participants’ characteristics are presented in Table 2.

**Table 2:** Sociodemographic and professional characteristics of Primary Health Care physicians working in municipalities with higher and lower endemicity

Sociodemographic and professional profile	HEM^a^		LEM^b^	
	*n*	(%)	*n*	(%)
Sex				
Male	4	(67%)	9	(42%)
Female	2	(33%)	12	(57%)
State of graduation				
Minas Gerais	4	(67%)	17	(81%)
Rio de Janeiro	1	(17%)	3	(14%)
Mato Grosso do Sul	0	(0%)	1	(5%)
Pernambuco	1	(17%)	0	(0%)
Postgraduate training				
Yes, completed	4	(67%)	5	(24%)
Yes, ongoing	0	(0%)	4	(19%)
No	2	(33%)	12	(57%)
Length of experience in PHC^c^				
More than 5 years	3	(50%)	4	(19%)
1 to 5 years	2	(33%)	11	(52%)
6 to 12 months	0	(0%)	3	(14%)
Less than 6 months	1	(17%)	3	(14%)

^a^ HEM, higher-endemicity municipalities; ^b^ LEM, lower-endemicity municipality; ^c^ PHC, Primary Health Care

Three thematic categories emerged from the data analysis, each comprising several meaning units, as presented in Table 3.

**Table 3:** Thematic categories and corresponding meaning units

Thematic categories	Meaning units
Professional training	Gaps in medical education and professional training on Chagas disease
Clinical management	Weaknesses identified in diagnosis and treatment
Physicians’ perceptions and knowledge regarding Chagas disease	Challenges in recognizing and diagnosing Chagas disease
	The invisibility of Chagas disease within health services and the community

### Gaps in Medical Education and Professional Training on CD

#### Professional Training

Overall, physicians from both the HEM and the LEM reported that CD had been addressed predominantly from a theoretical perspective during undergraduate medical education.

“During medical school, we only had the ‘textbook patient.’ The ‘textbook patient’ is great because they present all the symptoms, including skin lesions.” P15LEM.

Some participants, however, reported having encountered individuals with CD during clinical training and emphasized that these experiences mainly involved patients with complications associated with the chronic form of the disease.

“When patients with CD show up during medical school, they are usually the more dramatic cases. That really leaves an impression. The first time you see a megaesophagus, you see the patient in an emergency situation, severely congested. You think, ‘Wow, this is what CD looks like.’ So, it is something very memorable.” P19LEM.

Physicians also reported that they had not received any continuing education (CE) activities related to CD in their municipalities.

“We have not received any training on CD.” P8LEM.

### Weaknesses Identified in Diagnosis and Treatment

#### Clinical Management

Physicians demonstrated uncertainty and limited knowledge regarding updates in diagnostic methods for CD.

“Do you still hear anything about the Machado-Guerreiro test?” P1HEM.

In the HEM, physicians reported that serological testing is rarely requested because most patients receiving routine PHC care already have a confirmed diagnosis.

“We have not seen any patients presenting with symptoms that would lead us to request serological testing; they usually come to us with a previous diagnosis.” P1HEM.

Participants also highlighted the epidemiological context as an important factor guiding diagnostic suspicion.

“Diagnostic suspicion and the additional tests we request to establish the diagnosis are driven more by epidemiological factors than by symptoms.” P5HEM.

Physicians from the LEM reported that CD was often not considered among the initial diagnostic hypotheses, even in clinical situations compatible with the disease.

“A patient came in with severe congestion and cardiomegaly. He was originally from Northern Minas, had already been followed by another specialty, and no one had suspected CD. By the time he came to me, he was already in heart failure. We receive a large number of patients from Northern Minas. That case was a wake-up call for me because, before that, CD would probably have been the third or fourth diagnosis I would consider if I saw a bundle branch block.” P15LEM.

In addition, physicians from all municipalities reported uncertainties and divergent practices regarding the notification of chronic CD.

“We report dengue and COVID-19 cases, but I had never heard that CD should also be reported.” P1HEM.

“The place where I used to work was a little ahead in this regard because we used to report CD cases.” P3HEM.

Physicians reported that their knowledge regarding BZN prescription was derived exclusively from their undergraduate medical training. Overall, participants stated that they were aware of the indications and benefits of BZN treatment only during the acute phase and were unaware of its potential benefits during the chronic phase of CD.

“I still rely on what I learned in medical school. We do not treat chronic cases with BZN.” P7LEM.

“As far as I know, BZN is indicated during the acute phase, within the first four weeks. In the chronic phase, it has no benefit.” P6HEM.

“I know that antiparasitic medication exists mainly for the acute phase, but I have never prescribed it.” P23LEM.

Physicians emphasized that the main barrier to prescribing BZN was the lack of updated knowledge regarding current treatment recommendations. They also highlighted the need for training activities aimed at updating their knowledge on antiparasitic treatment for CD.

“The difficulty lies in keeping up to date. We learned during medical school that BZN was used only in the acute phase, and because that knowledge remained unchanged, this has become a gap in our professional updating.” P3HEM.

“It is really a matter of insecurity because we do not know how the guidelines have changed. A lot has changed, so we need to keep ourselves updated. Currently, I do not feel confident prescribing BZN, and this is a different kind of insecurity from what I experienced when I first graduated. What is missing is training or an update on BZN.” P1HEM.

“If we are properly instructed on dispensing procedures and referral pathways, we will have no difficulty prescribing BZN.” P6HEM.

When asked about the CPTG for CD, physicians who responded reported being unfamiliar with this guideline.

“No, not the 2018 guideline you mentioned.” P5HEM.

### Challenges in Recognizing and Diagnosing CD

#### Physicians’ Perceptions and Knowledge Regarding CD

Some physicians from the LEM reported that the slow progression of chronic CD may lead patients to delay seeking medical care.

“Because chronic CD may take decades to produce symptoms, patients tend to put off seeking care. When patients are vomiting, they go to the health unit. However, when they notice that they become slightly short of breath while walking, they may think they are simply tired because they work too much or are just feeling fatigued. As a result, patients keep postponing seeking care because of the slow progression of the disease.” P18LEM.

Participants also reported that the absence of evident clinical manifestations makes the diagnosis of CD more difficult, and the disease is not always considered among the initial diagnostic hypotheses.

“When the disease is already advanced, making the diagnosis is very easy. You can even suspect it from a chest X-ray because, with a heart that enlarged, it is hard not to think about CD. However, when the patient presents only with intestinal dysfunction or other less obvious manifestations, which is the most difficult situation, I think diagnosing it becomes very challenging.” P24LEM.

“When patients present with more severe manifestations, the diagnosis becomes clearer. However, when the patient is younger, the diagnosis often goes unnoticed. In an older patient with a classic clinical history, it is much easier.” P19LEM.

“Despite our clinical assessment at the time of the consultation, CD is not among our first diagnostic considerations.” P8LEM.

#### The Invisibility of CD Within Health Services and the Community.

Physicians highlighted that many patients have limited understanding of the progression of CD and of the clinical relevance of its associated symptoms, which contributes to delays in seeking medical care.

“For him, he is a cardiac patient. He does not see himself as a patient with CD. He does not make that connection.” P24LEM.

“They do not recognize the severity of the disease. For example, when there is cardiac involvement, they do not realize that the shortness of breath they are experiencing is serious. As a result, they do not seek care at the health unit. Only those with more severe disease perceive things somewhat differently, but even then, they often resist acknowledging the severity of their condition. Some remain at home with decompensated atrial fibrillation and chest discomfort without seeking care at the health unit.” P5HEM.

In the HEM, physicians emphasized that CD receives limited attention in routine health care practices because it is not linked to indicators or targets established by the Federal Government.

“In the end, there is a strong focus on meeting the Federal Government’s targets, and it becomes difficult to prioritize other issues because CD is not included among those targets.” P5HEM.

In the LEM, some physicians perceived that CD is not recognized as a relevant public health problem.

“Our perspective is not really focused on CD as a public health problem that needs to be addressed.” P7LEM.

## Discussion

This study identified challenges related to the management of CD by PHC physicians working in HEM and LEM in MG, Brazil. The findings revealed weaknesses in professional training, technical and scientific updating, the incorporation of clinical guidelines, and the integration between PHC and Health Surveillance. Taken together, these weaknesses suggest that CD remains a low-priority condition within health services. This scenario may compromise the early identification of cases, the timely provision of treatment, and the production of reliable information for disease surveillance and monitoring.

Physicians’ reports indicated that education on CD was predominantly theoretical and poorly integrated into clinical practice, particularly within the PHC setting [6, 11]. This finding is not fully consistent with the Brazilian National Curricular Guidelines for Medical Education [20], which advocate training aligned with the population’s health needs, integrated across different levels of care, and responsive to the epidemiological characteristics of local contexts. Exposure to patients with CD occurred predominantly in situations involving complications of the chronic form, being largely restricted to more severe clinical manifestations. This educational model may reinforce the recognition of advanced disease presentations while limiting the identification of asymptomatic or early-stage cases, precisely when antiparasitic treatment offers the greatest clinical benefit [9]. These findings corroborate previous evidence of persistent gaps in medical education regarding both PHC needs and the management of CD [6, 11]. This scenario may reflect the fragmentation of the health care network, characterized by barriers to access and weaknesses in coordination across levels of care [21, 22]. In this context, care tends to be concentrated in specialized services [23], increasing medical students’ exposure to patients with more advanced stages of the disease.

The weaknesses identified in undergraduate medical education appear to persist throughout professional practice. The absence of CE activities on CD, even in municipalities with higher endemicity, suggests difficulties in organizing educational processes that are aligned with the needs of health care practice and the local epidemiological context [24, 25]. This issue is particularly critical for CD, as its diagnosis depends largely on clinical suspicion and epidemiological investigation [9]. The Brazilian National Policy on Continuing Health Education recognizes workplace-based learning as a central strategy for improving professional practice [25]. However, the findings indicate that CD still does not occupy a prominent place in local professional development strategies.

In this context, the lack of familiarity with the CPTG for CD deserves particular attention. This document provides guidance for the management of CD in PHC by offering systematized recommendations for the diagnosis, treatment, and clinical follow-up of affected individuals [9]. Considering that the participants were working in territories with a known presence of the disease, their limited familiarity with these guidelines is particularly noteworthy. The limited uptake of the CPTG suggests weaknesses in the dissemination and incorporation of technical and scientific recommendations into routine health care practice. This scenario also highlights the shared responsibility of health professionals for maintaining up-to-date knowledge, particularly in light of the uncertainties reported regarding disease management. In this context, the use of technical references and normative documents may contribute to updating professional knowledge and improving clinical practice.

Limited knowledge of updated diagnostic methods for CD may contribute to the underdiagnosis of the disease, considering that approximately 70% of affected individuals remain undiagnosed [26–28]. Although the CPTG provide updated diagnostic recommendations [9], their availability alone does not ensure their incorporation into clinical practice. The limited incorporation of these recommendations suggests weaknesses in health services’ response to CD. As a consequence, difficulties in the early detection of cases and the timely provision of care to affected individuals may persist.

The low frequency of serological testing further reinforces this scenario. The perception that most patients already have an established diagnosis suggests a tendency to view CD as a condition limited to previously identified cases. This dynamic may reduce the incorporation of diagnostic investigation into routine clinical practice, thereby contributing to the persistence of underdiagnosis [28]. As a consequence, affected individuals may remain without access to timely treatment and follow-up care [29]. These findings underscore the need to recognize early detection as a strategic component of the response to CD within PHC.

Weaknesses in technical and scientific updating also have implications for the provision of antiparasitic treatment. Knowledge regarding BZN that remains restricted to undergraduate medical education indicates difficulties in incorporating changes in therapeutic recommendations for CD into clinical practice [9, 30]. Gaps in knowledge regarding the indications and benefits of BZN, both in the acute and chronic phases of the disease, further reinforce this finding. This scenario may hinder the use of BZN in PHC, limiting patients’ access to a recommended intervention for the management of CD. In this context, strengthening PHC capacity for the therapeutic management of the disease may contribute to expanding access to antiparasitic treatment [9, 28, 29].

Uncertainties regarding the need to notify cases of CD suggest that surveillance activities have not yet been fully incorporated into routine PHC practice. This scenario may compromise the epidemiological monitoring of the disease, for which case notification constitutes an essential tool [31]. This finding is particularly relevant given the strategic role of PHC in generating information to support Health Surveillance activities [8, 31]. Despite the recent implementation of mandatory notification for chronic CD and the availability of a specific reporting tool within the e-SUS Notifica system [32, 33], uncertainties and heterogeneous notification practices persist. This situation suggests that regulatory implementation has not been accompanied by sufficient strategies to incorporate these recommendations into routine work processes. As a consequence, weaknesses in the integration between PHC and Health Surveillance may persist, limiting case detection and reporting within health information systems.

Challenges related to the recognition of CD also involve aspects inherent to its natural history. The slow progression of the disease and its often nonspecific manifestations may contribute to the normalization of early symptoms and hinder their recognition as indicators of illness [34–37]. As a consequence, both patients and health professionals may underestimate the clinical significance of these manifestations, delaying, respectively, the search for medical care and the formulation of diagnostic hypotheses. In this context, the systematic incorporation of epidemiological information into clinical reasoning becomes particularly important for increasing diagnostic suspicion and facilitating the identification of individuals presenting with nonspecific manifestations [38].

Physicians’ reports also suggest that patients experience difficulties in understanding the implications of CD for their own health. The dissociation between symptoms, disease progression, and diagnosis may compromise risk perception and hinder understanding of the disease and its consequences, which is consistent with the concept of health literacy [39]. This scenario may impair patients’ engagement in longitudinal follow-up and disease-related self-care practices [36, 40]. Therefore, health education interventions targeting the population play an important role in increasing knowledge about CD and promoting individuals’ active participation in their own care.

Another aspect that deserves attention concerns the precarious nature of employment arrangements. The absence of recruitment through civil service examinations among all participants may contribute to professional turnover, a phenomenon frequently associated with the search for better working conditions and remuneration [41, 42]. This dynamic may compromise continuity of care and the establishment of long-term relationships with the population, both of which are core attributes of PHC [43]. In the context of CD, such continuity is particularly important for patient follow-up and care coordination [9, 28].

The invisibility of CD is also reflected in the organization of health services and management agendas. The absence of the disease from priority targets and indicators may hinder its incorporation into health planning, monitoring, and service organization processes. As a consequence, CD tends to receive lower priority compared with other conditions already established within these agendas, contributing to the persistence of its institutional invisibility [37].

Taken together, the findings suggest that the invisibility of CD stems from interdependent weaknesses across different dimensions of health care. Higher local endemicity alone was not sufficient to ensure the incorporation of CD into routine PHC work processes. This finding indicates that geographical proximity to the disease, although relevant, does not replace systematic strategies aimed at professional development, strengthening CE, and enhancing integration between PHC and Health Surveillance. Addressing these weaknesses is therefore essential to strengthen PHC capacity to recognize and provide follow-up care for individuals affected by CD.

This study has some limitations that should be acknowledged. First, two of the four FG included a small number of participants, with two and four physicians participating, respectively. This configuration reflected the reality of the small municipalities investigated, which were characterized by a limited number of physicians working in PHC, and did not preclude collective discussions capable of providing in-depth insights into experiences and perceptions related to the management of CD.

## Conclusion

This study analyzed PHC physicians’ perceptions of challenges related to the management of CD in municipalities with higher and lower endemicity in MG, Brazil. Across both contexts investigated, the findings revealed weaknesses in professional training, CE, the incorporation of clinical guidelines, and the integration between PHC and Health Surveillance.

These limitations may compromise case identification, the provision of antiparasitic treatment, disease notification, and patient follow-up. Consequently, they contribute to maintaining the invisibility of CD within health services. Overcoming these challenges requires strengthening professional development, CE, and the integration between PHC and Health Surveillance, while taking into account the epidemiological and territorial specificities of municipalities. Studies including other PHC professionals may broaden the understanding of these challenges and support improvements in care and surveillance practices. In this process, making CD visible within health services is essential for its effective incorporation into PHC care and surveillance practices.

## Supplementary Information

Below is the link to the electronic supplementary material.

Supplementary Material 1

## Data Availability

All relevant data supporting the findings of this study are included in the manuscript and its Supplementary Information file.

## References

[1] *Global report on neglected tropical diseases 2024*. World Health World Health Organization, & Organization (2024). https://www.who.int/teams/control-of-neglected-tropical-diseases/global-report-on-neglected-tropical-diseases-2024

[2] Brasil. Ministério da Saúde Secretaria de Vigilância em Saúde. (2020). *Doença de Chagas: 14 de abril – Dia Mundial*. *Boletim Epidemiológico, 51*(Número especial), 1–43. https://www.gov.br/saude/pt-br/assuntos/saude-de-a-a-z/d/doenca-de-chagas/arquivos/be-numero-especial-doenca-de-chagas-14-de-abril-dia-mundial-2020.pdf

[3] Brasil (2020). Ministério da Saúde. Secretaria de Vigilância em Saúde. Coordenação-Geral de Informações e Análises Epidemiológicas. *Sistema de Informações sobre Mortalidade*. Ministério da Saúde. https://svs.aids.gov.br/daent/cgiae/

[4] Instituto Brasileiro de Geografia e Estatística (2010). *Censo demográfico 2010: Características da população e dos domicílios: Resultados do universo*. IBGE. https://censo2010.ibge.gov.br/

[5] Souza, A. B., Lacerda, A. M., Ferreira, A. M., Damasceno, R. F., Sabino, E. C., Ribeiro, A. L. P., Vieira, T. M., & Haikal, D. S. (2021). Estudo longitudinal de indivíduos com doença de Chagas de região endêmica brasileira: A coorte Sami-Trop. *Revista Unimontes Científica*, *23*(2), 1–22.

[6] Damasceno, R. F., Sabino, E. C., Ferreira, A. M., Ribeiro, A. L. P., Moreira, H. F., Prates, T. E. C., Sampaio, C. A., & Haikal, D. S. (2020). Challenges in the care of patients with Chagas disease in the Brazilian public health system: A qualitative study with primary health care doctors. *PLoS Neglected Tropical Diseases*, *14*(11), e0008782. 10.1371/journal.pntd.0008782 PMC7676681 33166280

[7] Brasil Conselho Nacional de Secretários de Saúde. (2003). *Legislação do SUS*. CONASS. https://bvsms.saude.gov.br/bvs/publicacoes/progestores/leg_sus.pdf

[8] Brasil. Ministério da Saúde (2017, September 22). Portaria nº 2.436, de 21 de setembro de 2017. Aprova a Política Nacional de Atenção Básica, estabelecendo a revisão de diretrizes para a organização da Atenção Básica, no âmbito do Sistema Único de Saúde (SUS). *Diário Oficial da União*. https://bvsms.saude.gov.br/bvs/saudelegis/gm/2017/prt2436_22_09_2017.html

[9] Brasil. Ministério da Saúde (2018). *Protocolo Clínico e Diretrizes Terapêuticas da Doença de Chagas*. Ministério da Saúde. https://www.gov.br/conitec/pt-br/midias/protocolos/pcdt_doenca_de_chagas.pdf

[10] Pan American Health Organization (2019). *Guidelines for the diagnosis and treatment of Chagas disease*. Pan American Health Organization. https://iris.paho.org/items/f1ca50c2-7c95-4251-9cec-cd1860e23a36

[11] Ferreira, A. M., Sabino, E. C., Moreira, H. F., Cardoso, C. S., Oliveira, C. D., Ribeiro, A. L. P., Ramos, B. C., & Haikal, D. S. (2018). Avaliação do conhecimento acerca do manejo clínico de portadores da doença de Chagas em região endêmica no Brasil. *Revista APS*, *21*, 345–354. 10.34019/1809-8363.2018.v21.16230

[12] Brasil. Conselho Nacional de Saúde (2013, June 13). Resolução nº 466, de 12 de dezembro de 2012. Aprova diretrizes e normas regulamentadoras de pesquisas envolvendo seres humanos. *Diário Oficial da União*. https://www.gov.br/conselho-nacional-de-saude/pt-br/atos-normativos/resolucoes/2012/resolucao-no-466.pdf/view

[13] Minayo, M. C. S. (2014). *O desafio do conhecimento: Pesquisa qualitativa em saúde* (14ª ed.). Hucitec.

[14] Flick, U. (2008). *Introdução à pesquisa qualitativa* (3ª ed.). Artmed.

[15] Kitzinger, J. (1994). The methodology of focus groups: The importance of interaction between research participants. *Sociology of Health & Illness*, *16*(1), 103–121. 10.1111/1467-9566.ep11347023

[16] Tong, A., Sainsbury, P., & Craig, J. (2007). Consolidated criteria for reporting qualitative research (COREQ): A 32-item checklist for interviews and focus groups. *International Journal for Quality in Health Care*, *19*(6), 349–357. 10.1093/intqhc/mzm042 17872937

[17] Eccles, M. P., & Mittman, B. S. (2006). Welcome to implementation science. *Implementation Science*, *1*, 1. 10.1186/1748-5908-1-1

[18] Campos, C. J. G., & Saidel, M. G. B. (2022). Amostragem em investigações qualitativas: Conceitos e aplicações ao campo da saúde. *Revista Pesquisa Qualitativa*, *10*(25), 404–412. 10.33361/RPQ.2022.v.10.n.25.545

[19] Bardin, L. (1977). *Análise de conteúdo*. Edições 70.

[20] Brasil. Ministério da Educação (2014, June 23). Resolução nº 3, de 20 de junho de 2014. Institui Diretrizes Curriculares Nacionais do Curso de Graduação em Medicina e dá outras providências. *Diário Oficial da União*, Seção 1, 8–11. https://www.gov.br/saude/pt-br/acesso-a-informacao/acoes-e-programas/pnsp/legislacao/resolucoes/rces003_14.pdf/view

[21] Mendes, E. V. (2010). As redes de atenção à saúde. *Ciência & Saúde Coletiva*, *15*(5), 2297–2305. 10.1590/S1413-81232010000500005 20802863

[22] Silva, A. B., Barroso, D. C. B. S. B., Fonseca, S. S. S., Freitas, S. P. C., Santos, J. P., & Mallet, J. R. (2024). Entraves que permeiam a Atenção Primária à Saúde para o manejo e controle da doença de Chagas no Brasil. *Revista Eletrônica Acervo Saúde*, *24*(7), e17773. 10.25248/REAS.e17773.2024

[23] Almeida, P. F., Giovanella, L., Mendonça, M. H. M., & Escorel, S. (2010). Desafios à coordenação dos cuidados em saúde: Estratégias de integração entre níveis assistenciais em grandes centros urbanos. *Cadernos de Saúde Pública*, *26*(2), 286–298. 10.1590/S0102-311X2010000200008 20396844

[24] Ceccim, R. B., & Feuerwerker, L. C. M. (2004). O quadrilátero da formação para a área da saúde: Ensino, gestão, atenção e controle social. *Physis: Revista de Saúde Coletiva*, *14*(1), 41–65. 10.1590/S0103-73312004000100004

[25] Brasil Ministério da Saúde. (2004, February 13). Portaria GM/MS nº 198, de 13 de fevereiro de 2004. Institui a Política Nacional de Educação Permanente em Saúde. https://www.gov.br/saude/pt-br/composicao/sgtes/pneps

[26] Bern, C. (2015). Chagas’ disease. *New England Journal of Medicine*, *373*(5), 456–466. 10.1056/NEJMra1410150 26222561

[27] Echeverría, L. E., Marcus, R., Novick, G., Sosa-Estani, S., Ralston, K., Zaidel, E. J., Forsyth, C., Ribeiro, A. L. P., Mendoza, I., Falconi, M. L., Mitelman, J., Morillo, C. A., Pereiro, A. C., Pinazo, M. J., Salvatella, R., Martinez, F., Perel, P., Liprandi, Á. S., Piñeiro, D. J., & Molina, G. R. (2020). WHF IASC Roadmap on Chagas disease. *Global Heart*, *15*(1), 23. 10.5334/gh.484 PMC7218776 32489799

[28] Ferreira, R. A., & Diotaiuti, L. G. (2021). Chagas disease and primary health care in Brazil. *Journal of Biomedical Research & Environmental Sciences*, *2*(6), 463–465. 10.37871/jbres1261

[29] Santos, F. L. N., Costa, V. M., & Silva, R. A. E. (2025). Chagas disease in Brazil: New challenges and perspectives for old problems. *Memórias do Instituto Oswaldo Cruz*, *120*, e240279. 10.1590/0074-02760240279 PMC12274067 40699036

[30] Dias, J. C. P., Ramos Jr., A. N., Gontijo, E. D., Luquetti, A., Shikanai-Yasuda,M. A., Coura, J. R., Torres, R. M., Melo, J. R. C., Almeida, E. A., Oliveira Jr.,W., Silveira, A. C., Rezende, J. M., Pinto, F. S., Ferreira, A. W., Rassi, A., Fragata Filho, A. A., Sousa, A. S., Correia Filho, D., Jansen, A. M., … Alves, R. V. (2016).II Consenso Brasileiro em Doença de Chagas, 2015. *Epidemiologia e Serviços de Saúde, 25*(Número especial), 7–86. 10.5123/S1679-49742016000500002. 27869914

[31] Brasil (2024). Ministério da Saúde. Secretaria de Vigilância em Saúde e Ambiente. *Guia de Vigilância em Saúde* (6ª ed. rev.). Ministério da Saúde. https://www.gov.br/saude/pt-br/centrais-de-conteudo/publicacoes/svsa/vigilancia/guia-de-vigilancia-em-saude-volume-1-6a-edicao/view

[32] Brasil. Ministério da Saúde (2020, May 29). Portaria nº 1.061, de 18 de maio de 2020. Inclui a doença de Chagas crônica na Lista Nacional de Notificação Compulsória de doenças, agravos e eventos de saúde pública. *Diário Oficial da União*. https://www.cevs.rs.gov.br/upload/arquivos/202111/05142728-portaria-n-1061-de-18-de-maio-de-2020.pdf

[33] Brasil (2024). Ministério da Saúde. Secretaria de Vigilância em Saúde e Ambiente. *Guia para notificação de Doença de Chagas Crônica (DCC)*. Ministério da Saúde. https://www.gov.br/saude/pt-br/centrais-de-conteudo/publicacoes/svsa/doenca-de-chagas/guia-para-notificacao-de-doenca-de-chagas-cronicas-dcc/view

[34] RassiJr, A., Rassi, A., & Marin-Neto, J. A. (2010). Chagas disease. *The Lancet*, *375*(9723), 1388–1402. 10.1016/S0140-6736(10)60061-X 20399979

[35] Pérez-Molina, J. A., & Molina, I. (2018). Chagas disease. *The Lancet*, *391*(10115), 82–94. 10.1016/S0140-6736(17)31612-4 28673423

[36] Ventura-Garcia, L., Roura, M., Pell, C., Posada, E., Gascón, J., Aldasoro, E., Muñoz, J., & Pool, R. (2013). Socio-cultural aspects of Chagas disease: A systematic review of qualitative research. *PLoS Neglected Tropical Diseases*, *7*(9), e2410. 10.1371/journal.pntd.0002410 PMC3772024 24069473

[37] Pereira-Silva, F. S., Mello, M. L. B. C., & Araújo-Jorge, T. C. (2022). Doença de Chagas: Enfrentando a invisibilidade pela análise de histórias de vida de portadores crônicos. *Ciência & Saúde Coletiva*, *27*(5), 1939–1949. 10.1590/1413-81232022275.08492021 35544821

[38] Organização Pan-Americana da Saúde (2019). *Guia para diagnóstico e tratamento da doença de Chagas*. OPAS. https://www.paho.org/pt/noticias/7-1-2019-opas-divulga-novo-guia-para-diagnostico-e-tratamento-da-doenca-chagas

[39] Sorensen, K., Van den Broucke, S., Fullam, J., Doyle, G., Pelikan, J., Slonska, Z., Brand, H., & HLS-EU) Consortium Health Literacy Project European. (2012). Health literacy and public health: A systematic review and integration of definitions and models. *Bmc Public Health*, *12*, 80. 10.1186/1471-2458-12-80 PMC3292515 22276600

[40] Baldoni, N. R., Quintino, N. D., Ferreira, A. M., da Silva, J. L. P., Ribeiro, A. L. P., Oliveira, C. D. L., Sabino, E. C., & Cardoso, C. S. (2024). Health literacy assessment of individuals with and without Chagas disease: A cross-sectional study. *BMC Infectious Diseases*, *24*(1), 1414. 10.1186/s12879-024-10213-6 PMC11653836 39695986

[41] Barros, R. D., Melo, H. C., & Soares, R. L. C. (2026). Análise dos vínculos trabalhistas e tempo de permanência dos profissionais da atenção primária no Brasil. *Saúde em Debate*, *50*(148), e10701. 10.1590/2358-2898202614810701P

[42] Campos, C. V. A., & Malik, A. M. (2008). Satisfação no trabalho e rotatividade dos médicos do Programa de Saúde da Família. *Revista de Administração Pública*, *42*(2), 347–368. 10.1590/S0034-76122008000200007

[43] Starfield, B. (2002). *Atenção Primária: Equilíbrio entre necessidades de saúde, serviços e tecnologia*. UNESCO/Ministério da Saúde.

