# Supplementary material for: Management of Chagas Disease in Primary Health Care: Challenges Faced by Physicians in Areas with Higher and Lower Endemicity in Brazil
Source: J Community Health. 2026 Jul 23;51(5):939–48. doi: 10.1007/s10900-026-01595-6 (PMC13615010; doi:10.1007/s10900-026-01595-6)
Supplement: Supplementary file 1 [file 10900_2026_1595_MOESM1_ESM.docx]

**MANAGEMENT OF CHAGAS DISEASE IN PRIMARY HEALTH CARE: CHALLENGES FACED BY PHYSICIANS IN AREAS WITH HIGHER AND LOWER ENDEMICITY IN BRAZIL**

**JOURNAL OF COMMUNITY HEALTH**

Andréia Brito de Souza^1*^; Denise Alves Guimarães^2^; Renata Fiuza Damasceno^3^; Mayra Domingues Cardoso^4^; Ariela Mota Ferreira^1^; Clareci Silva Cardoso^2^; Ester Cerdeira Sabino^5^; Desirée Sant´Ana Haikal^1^

^1^ Graduate Program in Health Sciences, State University of Montes Claros, Montes Claros, Minas Gerais, Brazil

^2^ School of Medicine, Federal University of São João del-Rei, Divinópolis, Minas Gerais, Brazil

^3^ Regional Health Superintendence of Divinópolis, Minas Gerais State Health Department (SES-MG), Divinópolis, Minas Gerais, Brazil

^4^ State University of Montes Claros, Montes Claros, Minas Gerais, Brazil

^5^ Institute of Tropical Medicine, University of São Paulo, São Paulo, São Paulo, Brazil

***** Corresponding author

E-mail: [brito.ad2014@gmail.com](mailto:brito.ad2014@gmail.com)

**Online Resource 1.** Complete statements in Portuguese from primary healthcare physicians cited in the manuscript.

**Table 1**. Anonymized participant statements

**Professional training**

*Gaps in medical education and professional training on CD*

“Na graduação, só “paciente de livro”. Então assim, é tudo de paciente de livro, paciente de livro é ótimo, porque vem todos os sintomas né, até lesões de pele, assim” P15LEM

“E como o paciente chagásico quando vê na faculdade já é daqueles casos mais escandalosos, aí isso é bem marcante, então quando você vê um megaesôfago pela primeira vez, você vê na prática o paciente com urgência, todo congesto. Nossa, isso aí que é Chagas. Então, é algo marcante. E mesmo na faculdade, quando você só vê ali alguns casos, é marcante” P19LEM

“Então eu também, eu não tenho vivência nenhuma, nem informação sobre o tratamento da forma crônica, a gente não teve uma capacitação sobre isso” P8LEM

**Clinical management**

*Weaknesses Identified in Diagnosis and Treatment*

“E aquele exame Machado Guerreiro, vocês têm notícia dele?” P1HEM

“Eu diagnosticar algum, ainda não, não chegou nenhum com a clínica para poder pedir a sorologia, já vieram com o diagnóstico pronto” P1HEM

“A suspeita e os exames extras que pedem para poder dar o diagnóstico é voltado para a parte epidemiológica, mais do que sintomas” P5HEM

“Estava investigando um paciente que chegou para mim todo congesto, com cardiomegalia. Ele é do norte de Minas, acompanhando com outra especialidade e ninguém suspeitou de Chagas e aí, na hora que chegou para mim, ele já estava com insuficiência cardíaca. A gente recebe uma população do norte de Minas muito grande, eu acho que agora me deu um start, porque para mim antes era, sei lá, terceira, quarta possibilidade que eu ia pensar se eu visse um bloqueio de ramo” P15LEM

“Notifica dengue, notifica COVID, mas eu nunca vi falar que tem notificação de Chagas.” P1HEM

“O lugar que eu trabalhava tava um pouco à frente disso porque a gente notificava.” P3HEM

“Eu vim com essa carga da faculdade, né? O caso crônico a gente não trata” P7LEM

“Eu sei que a indicação dele seria na fase aguda, nas primeiras quatro semanas, na fase crônica não tem benefício” P6HEM

“Eu sei que existe medicação principalmente para fase aguda, mas nunca prescrevi” P23LEM

“A dificuldade é na atualização. Como a gente estudou e deixamos lá esse conhecimento que usaria na fase aguda. Seria mesmo uma falha na atualização de conhecimento” P3HEM

“É questão da insegurança mesmo, não saber como mudou esse protocolo. Aí vai vir aquela insegurança porque mudou muita coisa, então a gente tem que estar atualizando. Hoje, eu estou inseguro em medicar, e não é a mesma insegurança que eu tinha na época em que formei. Está faltando uma capacitação ou então uma atualização, mandar pelo menos um órgão.” P1HEM

“Tenho segurança de que sim, a gente sendo orientado como a dispensação e os fluxos não têm dificuldade não, né.” P6HEM

“O que você falou de 2018 não.” P5HEM

**Physicians’ perceptions and knowledge of CD**

*Challenges in Recognizing and Diagnosing CD*

“E a chagas crônica, por ser assim, décadas para desenvolver os sintomas do paciente, mesmo ele vai marinando. O paciente procura muito mais o PSF porque está com uma dor na coluna pelo trabalho, porque o meu chefe me cobra de mais, não aguento ficar naquele trabalho mais, minha coluna me mata. Procura muito mais isso do que um sintoma que é gradual, que é lento. Que não acontece do dia para a noite. O paciente, quando ele está, ah, estou vomitando, ele vai para o PFS. Agora, quando é aquilo lá, pensando, estou ficando um pouquinho cansado quando eu ando, ah, talvez é eu estou indisposto, trabalho demais, cansado. Aí o paciente vai, vai marinando por causa do tempo também da evolução da doença.” P18LEM

“Quando está crônico é muito fácil fazer o diagnóstico, você faz até pelo Raio-x porque um coração daquele tamanho acho que não tem como você não pensar em Chagas, agora quando está só com uma disfunção intestinal ou uma coisa assim não tão nítida, que é a parte mais difícil, eu acho que é muito difícil de diagnosticar.” P24LEM

“Acho que depende muito do sintoma que o paciente está apresentando, quando chega um caso mais exacerbado, o diagnóstico fica mais nítido, agora quando é um paciente mais jovem, aí o diagnóstico acaba passando, um senhor com uma história toda bonitinha, aí é bem mais fácil” P19LEM

“Apesar do olhar clínico, nosso ali, no momento, não está entre as primeiras opções, de suspeita” P8LEM

*The Invisibility of CD Within Health Services and the Community*

“Mas para ele, ele é um paciente cardiopata, ele não é um paciente chagásico, entendeu? Ele não associou assim” P24LEM

“Eles não valorizam a gravidade, quando, por exemplo, tem acometimento cardíaco, eles não valorizam que uma falta de ar que eles estão sentindo é grave, aí eles não vão procurar. "Só quem está com a doença grave vê um pouquinho diferente, mesmo assim, eles têm uma resistência a notar que é grave, eles estão em casa com a fibrilação atrial descompensada, com incômodos torácicos, alguns não vão à unidade” P5HEM

“Destaque tem, agora eles têm as próprias metas do governo federal. Então, acaba que foca muito nas metas que tem que bater e fica um pouco difícil deslocar para outro assunto, porque chagas não entra dentro das metas” P5HEM

“Aqui, nosso olhar não é tão voltado como um problema de saúde pública que tem que ser combatido, tem que ser identificado” P7LEM
